# Effectiveness and Safety of Immune Checkpoint Inhibitors in Older Cancer Patients

**DOI:** 10.3390/jpm14030278

**Published:** 2024-03-01

**Authors:** Damir Vucinic, Iva Skocilic, Marin Golcic, Renata Dobrila-Dintinjana, Maja Kolak, Ivona Jerkovic, Eleonora Cini Tesar, Ani Mihaljevic Ferari, Arnela Redjovic, Jasna Marusic, Doris Kolovrat, Ivana Mikolasevic

**Affiliations:** 1 Tumor Clinic, Clinical Hospital Center Rijeka, 51000 Rijeka, Croatia; damir.vucinic@gmail.com (D.V.); iskocilic@gmail.com (I.S.); marin.golcic@gmail.com (M.G.); renatadobrila@windowslive.com (R.D.-D.); kolak.maja@gmail.com (M.K.); ivona.jerkovic051@gmail.com (I.J.); ecinitear@yahoo.com (E.C.T.); amferari15@gmail.com (A.M.F.); redzovica@gmail.com (A.R.); jasnaskrobonja@yahoo.com (J.M.); doris.kolovrat5@gmail.com (D.K.); 2 Faculty of Medicine, University of Rijeka, 51000 Rijeka, Croatia; 3 Department of Gastroenterology, Clinical Hospital Center Rijeka, 51000 Rijeka, Croatia

**Keywords:** immune-related adverse events, immunotherapy, octogenarians, older patients, overall survival, progression-free survival

## Abstract

Background: The development of immunotherapy checkpoint inhibitors (ICIs) has revolutionized cancer care. However, old patients are underrepresented in most clinical trials, although they represent a significant proportion of real-world patients. We aimed to evaluate the effectiveness and safety of ICIs in patients older than the age of 70. Methods: We performed a retrospective chart review of 145 patients aged 70 or older treated with ICIs for metastatic or unresectable cancer. Results: Median progression-free survival (PFS) was 10.4 months (95% CI 8.6–13.7), with no differences between octogenarians and septuagenarians (*p* = 0.41). Female gender (*p* = 0.04) and first-line treatment setting (*p* < 0.0001) were associated with a longer median PFS. Median overall survival (OS) was 20.7 months (95% CI 13.5–35.0 months), with no difference based on performance status, cancer site, gender, or between septuagenarians and octogenarians (all *p* > 0.005). Patients treated with ICIs in the first-line setting reported longer OS compared to treatment in the second-line setting (*p* < 0.001). Discontinuation of ICIs due to adverse effects was associated with both shorter PFS (*p* = 0.0005) and OS (*p* < 0.0001). Conclusion: The effectiveness of ICIs in older cancer patients primarily depends on the line of treatment and treatment discontinuation. Octogenarians experienced similar treatment responses, PFS, OS, and adverse effects compared to septuagenarians.

## 1. Introduction

Older age remains one of the most significant risk factors for many cancers. The median age at cancer diagnosis is 66 years, and the median age at cancer-related death is 72 years [1]. More than nine out of 10 cancers are diagnosed in people 45 and older. Those older than 74 make up almost 28% of all new cancer cases. As the world’s population ages, the number of older patients with cancer is increasing. In 2050, using GLOBOCAN, an estimated 6.9 million new cancers will be diagnosed in adults aged 80 years or older worldwide, up from 2.3 million in 2018 [2]. Globally, breast, lung, and colon were the most common cancer sites diagnosed in the oldest females, while prostate, lung, and colon were most frequent in the oldest males. The most usual definition of “older” people is aged 65, and it is the most commonly used cut-off to identify the older group of cancer patients in subgroup analyses in clinical trials, including immunotherapy [3]. However, some clinical trials use the age of 70 as a cut-off defining older people [3,4]. As the majority of people in their sixties currently maintain good health status, especially in developed countries, an older cut-off age might be more appropriate as a cut-off value [3].

The development of immunotherapy, particularly immune checkpoint inhibitors (ICIs), has revolutionized modern cancer care for patients of all ages. Immunotherapy is significantly more effective in treating cancer with fewer side effects compared to systemic chemotherapy [5]. Although immunotherapy enhances the immune system to treat cancer, it can also cause the immune system to damage normal, healthy tissue and cause serious immune-related adverse events. Inhibitors of cytotoxic T-lymphocyte-associated antigen 4 and programmed death receptor-1 (PD-1) and its ligand (PD-L1) are associated with improved overall survival (OS) for many tumor types with durable responses for a subset of patients [6]. Although these immunotherapy advances are promising for many patients with cancer, they also introduce new challenges for the care of older adults with cancer. In a large number of clinical trials regarding immunotherapy, older patients were underrepresented [3]. Older adults with cancer have a higher prevalence of comorbid conditions, functional impairments, and frailty, which may alter the efficacy and tolerability of ICIs. 

The immune system is gradually remodeled because of normal, physiologic aging. Aging implicates a remodeling of our immune system, which is a consequence of the physiological senescence of our cells and tissues coupled with environmental factors and chronic antigen exposure [7]. The alterations that affect the immune system during aging are termed immunosenescence, inflammaging, and cellular senescence. Immunosenescence is characterized by changes in the microenvironment of lymphoid organs such as the bone marrow and thymus, shifts in the relative abundance of immune cell subsets, and alterations in the makeup of circulating cytokines, which control immune homeostasis [6]. Aging of the bone marrow reinforces the hematopoietic stem cells into myeloid over lymphoid progenitors, resulting in the production of fewer immature T and B cells. Changes in the post-pubescent microenvironment of the bone marrow, thymus, and lymph nodes further compromise the maturation of these immature immune cells. Although peripheral signals initially maintain the circulating pool of antigen-inexperienced T cells in adults, encounters with environmental and self-antigens cause an increasing number of naive T cells to differentiate into effector and effector memory cells. One consequence of this expanding memory pool is a reduction of immunologic space, which causes a decrease in the T-cell repertoire and may limit the expansion of additional T-cell clones [6]. Due to immunosenescence, some data suggest that the effectiveness of ICIs can vary with respect to age, and it has been suggested that cancer patients who are older than 75 years have weaker responses in comparison to younger patients. Age-related shifts in circulating cytokines and chemokines, known as inflammaging, can also affect adaptive immunity. Inflammaging is the persistent, low-level activation of inflammatory responses in the absence of infection and is associated with morbidity and mortality among older adults [6]. Cellular senescence differs from immunosenescence. It is an age-related process that occurs in individual cells, rather than a combination of physiologic changes. Cellular senescence is an irreversible cell cycle arrest characterized by telomere attrition, epigenetic and metabolic rewiring, secretion of proinflammatory and matrix remodeling factors, and the persistent expression of cell-cycle inhibitors [6]. How they influence responses to immunotherapy is still unclear. Undoubtedly, intrinsic differences in the immune systems of older adults may influence the efficacy and/or toxicity of cancer immunotherapies.

Also, older adults are underrepresented in the large-scale clinical trials from which we derive efficacy and safety data for new cancer drugs, including ICIs [8]. Therefore, in the absence of prospective clinical trials, data collected from clinical practice can give us a good insight into the effectiveness and tolerance of ICIs in this group of patients, which is the aim of this paper.

## 2. Patients and Methods

### 2.1. Patient Population and Data Collection

We performed a retrospective review of electronic medical records of 145 patients who received nivolumab, pembrolizumab, durvalumab, or atezolizumab as therapy for metastatic or unresectable advanced cancers from September 2017 to December 2022 at Clinical Hospital Center (CHC) Rijeka. In our analysis, we used the age of 70 to define the elderly. The subgroup analysis investigated the differences in survival and incidence of side effects between septuagenarians (aged 70–79) and octogenarians (aged 80–89). Included patients had an Eastern Cooperative Oncology Group performance status (ECOG PS) of 0 or 1, measurable disease assessed with computerized tomography or magnetic resonance imaging per Response Evaluation Criteria in Solid Tumors 1.1 criteria, and an adequate organ function. Patients with central nervous system metastasis were included if asymptomatic or clinically controlled after whole-brain radiotherapy. We excluded patients with autoimmune diseases or other disorders requiring systemic immunosuppressive drugs, including corticosteroids (>10 mg daily prednisone or equivalent), a positive test for hepatitis B virus surface antigen or hepatitis C virus ribonucleic acid, and those testing positive for HIV. Patients were not required to undergo geriatric assessment.

### 2.2. Treatment

All enrolled patients received PD-1/PD-L1 inhibitor intravenously, according to a schedule of 10 mg/kg or 1500 mg every 2 weeks for durvalumab, 3 mg/kg or 240 mg every 2 weeks for nivolumab, 2 mg/kg or 200 mg every 3 weeks for pembrolizumab, and 1200 mg every 3 weeks for atezolizumab. Treatment was allowed to continue beyond progression or unacceptable toxicity or complication and up to a maximum of 24 months if the investigator considered that the clinical benefit to the patient persisted.

### 2.3. Evaluation of Side Effects of Immunotherapy

The detailed immune-related adverse event (irAE) profile and development of critical complications were evaluated. We assessed the development, severity, and clinical course of all irAEs: thyroid dysfunction, cutaneous disorders, interstitial pneumonitis, colitis, adrenal insufficiency, hepatitis, diabetes, and encephalitis. Hepatitis was defined as liver dysfunction with compatible pathological findings from liver biopsy or determined by a hepatologist. 

### 2.4. Outcomes and Measures

OS was defined as the time from the start of ICIs to death from any cause. Patients who were still alive at the time of data analysis were censored at the date of the last contact. Additional efficacy measures included progression-free survival (PFS)—the time from the start of ICIs to the disease progression or death date, whichever occurred first. The cut-off values were arbitrary and answered to an exploratory intention. Investigators made all treatment-based decisions using immune-related Response Criteria. However, we used the RECIST 1.1 criteria to determine PFS. We graded adverse events according to the NCI Common Terminology Criteria for Adverse Events version 4.0.

### 2.5. Statistical Analysis

Descriptive statistics were used to evaluate general data (including the median, average, quartile range, and standard deviation). The Kaplan–Meier method with the log-rank test was used for the survival analysis, while the Cox hazard ratio model (Cox regression) was used for the quantitative method of the ratio of the examined factors. *p* < 0.05 was used as the level of statistical significance. Data were processed using MedCalc Statistical Software version 14.8 (MedCalc Software, Ostend, Belgium) and Statistica 12 (StatSoft, Tulsa, OK, USA).

## 3. Results

### 3.1. General Data

The study included 145 consecutive patients with metastatic cancer aged 70 or older (range 70–86, median 74 years (95% CI 73–75)) treated with anti-PD1 or anti-PDL1 immunotherapy in the first- or second-line setting. A total of 19 patients (13.1%) were defined as octogenarians (80–86 years in our patient population). The majority of patients were male (*n* = 102, 70.3%), with ECOG status 1 (*n* = 119, 82.1%). The primary cancer site, type of immunotherapy, and the line of treatment are given in Table 1.

Regarding the most common cancer group (lung cancer), the majority of patients had adenocarcinoma (*n* = 62, 42.8% of the whole population), followed by squamous cancer (*n* = 27, 18.6%), non-specified type (*n* = 3, 2.1%), and microcellular (*n* = 1, 0.7%).

The majority of patients in the whole population were treated in the first-line setting (*n* = 88, 60.7%), while the rest were treated in the second-line setting (*n* = 57, 39.3%). While the majority of patients continued immunotherapy with no interruptions (*n* = 121, 83.4%), some patients either had treatment interruptions (*n* = 5, 3.4%; on average after 23.0 cycles (95% CI 12.5–33.5)) or permanent discontinuation of the treatment (*n* = 19, 13.1%; on average after 5.15 cycles (95% CI 2.75–7.57)) due to treatment side effects. There was no difference in age between the three groups (*p* = 0.055). A more detailed overview of adverse effects leading to treatment cessation or pause is given in Table 2. A total of 17 patients (11.7% of the study population) discontinued immunotherapy after 2 years of treatment with no progression, following the clause by the local governing body. The median number of applied immunotherapy cycles was 10 (95% CI 9–12, range 1–60).

The most common comorbidity was arterial hypertension (*n* = 97, 66.9%), followed by diabetes mellitus (*n* = 40, 27.6%), ischaemic heart disease (*n* = 16, 11.0%), and cerebrovascular insult (*n* = 9, 6.2%).

### 3.2. Progression-Free Survival

During the follow-up, 90 patients (62.1%) progressed or died. The group’s median PFS was 10.4 months (95% CI 8.6–13.7). 

**Figure 1:**
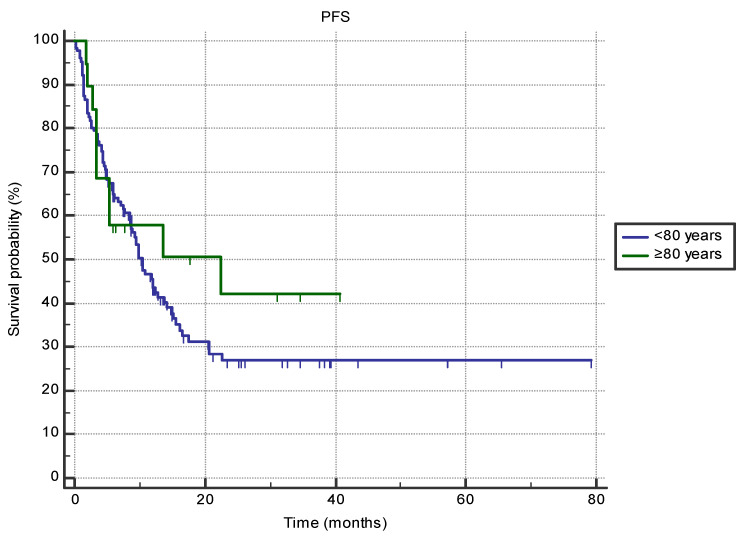
The difference in progression-free survival (PFS) between the groups of patients aged 80 or older (*n* = 19) and 70–80 (*n* = 126) treated with immune-checkpoint inhibitors in the first- or second-line setting (22.5 months, 95% CI 3.4–22.5 vs. 10.4 months, 95% CI 8.6–13.7, HR 0.76 (95% CI 0.42–1.38), *p* = 0.41).

There were no differences in PFS when comparing patients older than 80 with septuagenarians (22.5 months, 95% CI 3.4–22.5 vs. 10.4 months, 95% CI 8.6–13.7, HR 0.76 (95% CI 0.42–1.38), *p* = 0.41) (Figure 1). Survival between the two groups was not different based on the primary cancer site or ECOG status (all *p* > 0.05). However, female octogenarian patients exhibited longer PFS compared to male patients (N/r vs. 3.4 months (95% CI 3.3–13.5), *p* = 0.001), and patients treated in second-line setting exhibited longer PFS if they were over the age of 80 (22.5 months (95% CI 5.3–22.5) vs. 4.9 months (95% CI 2.3–8.6); *p* = 0.015).

We found no difference in PFS based on primary cancer location (*p* = 0.10), although a numerically longer PFS was found for melanoma patients (22.5 months, 95% CI 8.6–22.5) compared to lung cancer patients (9.9 months, 95% CI 7.5–13.7). Similarly, patients with lung adenocarcinoma had a numerically longer PFS compared to patients with squamous cell cancer (12.4 months, 95% CI 5.8–20.6 vs. 8.8 months, 95% CI 6.2–12.9, *p* = 0.20). Despite a small number of patients, a median PFS of less than 3 months was reported for kidney cancer patients (*n* = 6, 2.6 months, 95% CI 1.5–7.7) and bladder cancer patients (*n* = 6, 2.3 months, 95% CI 2.2–3.8).

Female patients exhibited a longer median PFS (13.7 months, 95% CI 9.8–15.1) compared to male patients (9.2 months, 95% CI 5.9–12.9) (*p* = 0.04), which persisted in lung cancer patients (*p* = 0.04), but not in only melanoma patients (*p* = 0.24).

Similarly, patients treated with immunotherapy in the first-line setting exhibited significantly longer PFS compared to patients treated in the second-line setting (15.5 months, 95% CI 10.7–22.7 vs. 5.3 months, 95% CI 3.3–9.2, *p* < 0.0001). The difference in PFS in the lines of treatment was mainly due to lung cancer group and particularly in patients with lung adenocarcinoma (22.7 months (95% CI 15.1–22.7) vs. 4.0 months (95% CI 2.0–9.2), *p* = 0.0002), while the difference was not significant in squamous cell lung cancers (9.9 months (95% CI 7.1–16.5) vs. 8.6 months (95% CI 2.0–13.5), *p* = 0.10).

**Figure 2:**
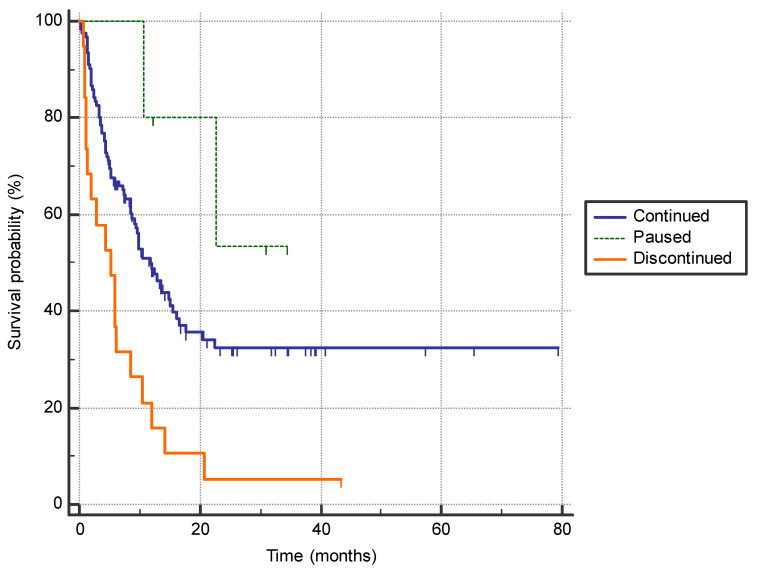
The difference in progression-free survival between the patients who permanently discontinued immunotherapy due to adverse effects (*n* = 19), who had a treatment pause with subsequent continuation of immunotherapy (*n* = 5), and patients who continued treatment with no significant adverse effects (*n* = 121) (5.3 months (95% CI 13.3–8.6) vs. not reached (95% CI n/a) vs. 11.8 months (95% CI 9.2–15.5); *p* = 0.0005).

Discontinuation of the immunotherapy due to the adverse effects was associated with a shorter PFS compared to patients who did not have adverse effects requiring treatment interruption or who only paused immunotherapy before continuation (5.3 months (95% CI 13.3–8.6) vs. 11.8 months (95% CI 9.2–15.5) vs. not reached (95% CI n/a) *p* = 0.0005)) (Figure 2).

### 3.3. Overall Survival

In total, 75 patients died during the follow-up (51.7%). The median OS for the whole group of patients was 20.7 months (95% CI 13.5–35.0 months).

**Figure 3:**
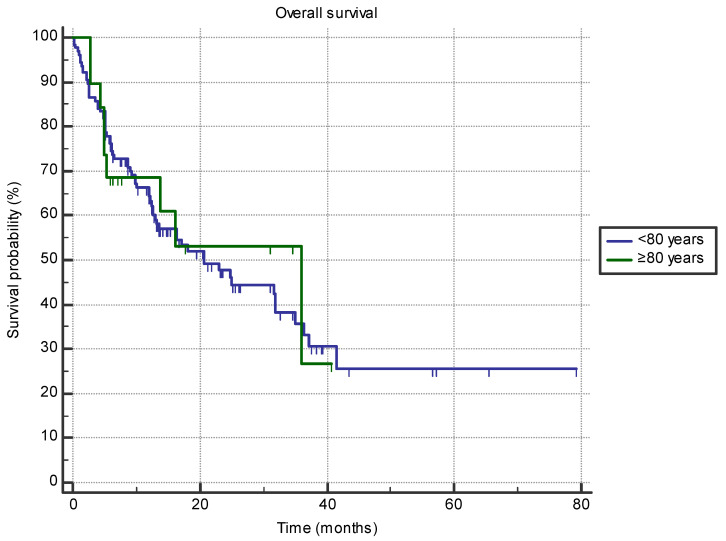
The difference in overall survival between the groups of patients aged 80 or older (*n* = 19) and 70–80 (*n* = 126) treated with immune-checkpoint inhibitors in the first- or second-line setting (35.9 months (95% CI 5.3–35.9 months) vs. 20.7 months (95% CI 13.2–31.9 months); HR 0.9, *p* = 0.79).

Octogenarian patients did not have shorter OS compared to septuagenarians (35.9 months (95% CI 5.3–35.9 months) vs. 20.7 months (95% CI 13.2–31.9 months); HR 0.9, *p* = 0.79) (Figure 3). No difference in OS between the two age groups was found for the line of treatment, ECOG status, primary cancer site, or gender (all *p* > 0.05).

There was no difference in OS when comparing the six most common types of cancer (*p* = 0.67), although melanoma patients exhibited numerically longer survival (35.9 months, 95% CI 13.2–41.5), compared to lung cancer patients (17.2 months, 95% CI 12.3–31.6) (*p* > 0.05). A similar trend was observed within the largest group of patients—the lung cancer patient group, where patients with adenocarcinoma (*n* = 62) reported longer OS compared to squamous cell cancer patients (*n* = 27) (24.9 months, 95% CI 9.2–37.2 vs. 16.1 months, 95% CI 9.9–18.2, *p* = 0.10).

Female patients exhibited longer OS compared to males (not reached vs. 16.3 months, *p* = 0.008). The difference in survival between the genders persisted when observing only the lung cancer group (*p* = 0.008), but was not reported for melanoma, the second-largest group of patients (*p* = 0.23).

Patients treated with immunotherapy in the first line of treatment reported longer OS (31.6 months, 95% CI 20.7–35.0) compared to treatment in second-line setting (12.6 months, 95% CI 5.3–24.7) (*p* < 0.001), while there was a trend towards longer OS in patients with ECOG 0, compared to ECOG 1 status (41.5 months, 95% CI 13.1–41.5 vs. 18.2 months, 95% CI 12.6–31.6; *p* = 0.09).

Similar to the data with PFS, longer OS with the first line of treatment, compared to the second line, was mainly due to lung cancer patients, particularly lung adenocarcinoma (35.0 months (95% CI 22.9–35.0) vs. 5.8 months (95% CI 5.1 vs. 24.7), *p* = 0.0020)). No significant difference between OS for the first- and second-line treatment was found for squamous cell lung cancer patients (16.2 months (95% CI 9.9–31.7) vs. 12.6 months (95% CI 5.1–16.3), *p* = 0.06).

**Figure 4:**
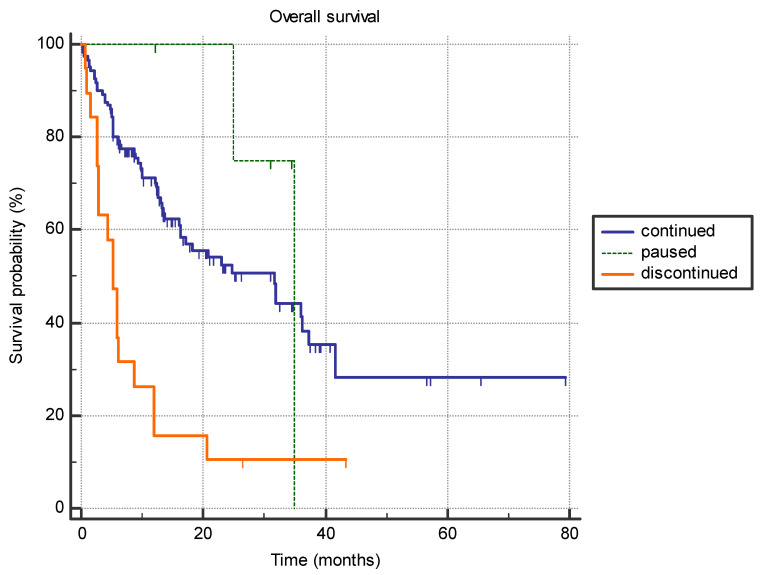
The difference in overall survival between the patients who permanently discontinued immunotherapy due to adverse effects (*n* = 19), patients who had a treatment pause with subsequent continuation of immunotherapy (*n* = 5), and patients who continued treatment with no significant adverse effects (*n* = 121) (5.3 months vs. 35.0 months vs. 31.6 months, *p* < 0.0001).

Patients who discontinued immunotherapy treatment due to side effects (*n* = 19), exhibited significantly shorter survival compared to patients who only exhibited a treatment pause (*n* = 5), or had no side effect and continued immunotherapy (*n* = 121) (5.3 months vs. 35.0 months vs. 31.6 months, *p* < 0.0001) (Figure 4).

## 4. Discussion

For now, there is no clear definition of an elderly in oncology. In most of the subgroup analyses of clinical trials, the age of 65 is set as a cut-off to define the elderly population. However, the age of 70 years is used as a cut-off to identify and screen elderly patients in the context of geriatric assessment dedicated to older cancer patients [9]. Therefore, in our analysis, we used the age of 70 to define the elderly. According to the FDA data of patients with cancer enrolled in clinical trials supporting registration in the 10-year period (from 2005 to 2015), 24% of included patients were 70 years or older, and only 12% were older than 75 [10]. There are two main potential dangers regarding the use of ICIs in older patients. Firstly, there is potential for higher toxicity as earlier data reported that the elderly population has a high prevalence of autoantibodies [11,12,13]. Secondly, changes in the immune system activity related to aging could influence treatment success as a decrease in proinflammatory activity of the adaptive and innate immune cells occurs with aging [12,13]. These issues raised doubts about potential ICI efficacy in elderly patients [13]. 

Geriatric syndrome, comorbidities, and organ dysfunction in older patients affect treatment outcomes and toxicity of therapy. Consequently, not as many older patients are included in studies, and there is not as much scientific evidence for treating them with ICIs. Therefore, selecting patients for ICI treatment depends on the clinicians’ assessment.

The meta-analysis by Kim et al. [14] showed no significant differences in the efficacy of ICI between patients below and above 65 years of age. In this meta-analysis, PFS was significantly higher only in younger melanoma patients. Other subgroups showed no difference. Melanoma is a highly immunogenic tumor so this PFS benefit might be connected to the decline of immune cells’ function [15,16,17,18,19,20]. The analysis of our results confirmed the data shown above. No significant differences in PFS or OS were demonstrated between patients older than 80 and in the 70–80-year-old subgroup in all cancer sites, but OS and PFS in patients with melanoma were numerically higher than in other cancer sites, regardless of age. None of the clinical studies showed better PFS or OS in geriatric patients [3,15,16,17,18,19,20]. Another meta-analysis [20] with 5000 patients showed no significant difference in PFS and OS between older and younger patients. It is well known that the efficiency of immunotherapy depends upon the patient’s immune system to activate anticancer effects. Therefore, our data and some earlier data suggest that some parts of the immune system maintain integrity even as we age [20,21,22], although further investigations on this topic are needed.

ICIs have a better toxicity profile compared to systemic chemotherapy, so they are potentially a suitable therapy choice for older patients, but quite often, they are not included in initiation studies. According to limited data, ICIs’ efficacy and toxicity among older patients with good performance status are comparable with toxicity and efficacy in younger patients [15,16,17,18,19,20,21,22].

Immunotherapy uses the body’s immune system to eliminate tumor cells but can cause the immune system to damage normal healthy cells, causing immune-related adverse events in all organs. Those side effects are rare but can be severe. Studies suggest similar toxicity profiles of ICIs in both younger and older patients, but older patients have worse outcomes if they do acquire side effects of grade 3 or 4 [6]. Older patients treated with corticosteroids are more likely to develop delirium, diabetes, osteoporosis, and opportunistic infections. Side effects of corticosteroid treatment likely aggravate older patients’ performance status and general condition [6,15,16,17].

We have limited information about potential risk factors for developing immunotherapy toxicity. According to data, few trials have focused on irAEs, specifically among older patients. Those studies reported similar incidence and severity of irAEs in older patients compared to younger adults. However, when analyzing these studies, comparisons regarding age were not always reported in the initial and post hoc 5-year clinical trial results [6,17,18,19,20,21]. Also, according to data, there are insufficient data on the severity and frequency of side effects with respect to age. In our cohort of patients, the most common side effects were hepatitis, followed by pneumonitis, colitis, and nephritis. In our study, a total of 13% of patients had permanent discontinuation of the treatment due to treatment side effects, which is consistent with other studies [18,19,20,21]. Perhaps unexpectedly, treatment discontinuation was associated with both shorter PFS and OS in our patient population, while the treatment interruption/pause cohort trended towards longer survival. While small patient groups could influence such results, a total of 7 out of 19 patients permanently terminated immunotherapy within 1 month, and an overlap between immunotherapy adverse effects and rapid disease progression could be another explanation of the results.

According to our results, female patients showed longer median PFS and median OS than male patients in both subgroups. Also, the difference was even more prominent in the subgroup of patients older than 80. For example, a large study by Morgese et al. also proved that women have a better survival rate than men in the case of locally advanced melanoma [23]. A total of 1023 patients who had been treated between 1987 and 2014 were enrolled in this big study. On the other hand, Conforti et al. evaluated patients treated with immunotherapy and reported OS regarding gender, suggesting that male patients derive greater benefits from immunotherapy [24]. A subsequent meta-analysis by Wallis et al. found no difference in efficacy between males and females [25].

In conclusion, according to our experience, septuagenarians and octogenarians had comparable treatment responses and survival rates, which is in line with earlier reported data. Similarly, the frequencies of side effects are comparable between the two age groups. These ‘real-world’ data are important because elderly patients are often not adequately represented in prospective clinical trials that evaluate the efficiency of ICIs. The decision to introduce ICIs depends on the clinician’s opinion. According to our experience, patients’ age does not represent a limitation for ICI introduction per se. However, the patient’s age should be considered as a main surrogate marker for evaluating other factors related to age, such as comorbidities, drug polytherapy, and decreased kidney function related to aging. Treating side effects is the same as in younger patients, but it is necessary to consider the potential side effects of corticosteroid treatment in elderly patients. 

## Figures and Tables

**Table 1:** Primary cancer site, number of patients, and type of immunotherapy used.

Primary Cancer Site	*n* (%)	Type of Immunotherapy Used (N)	Line of Treatment (N)	Gender, Female (N) (%)	Median Age, Years (95% CI)
Lung	93 (64.1)	Pembrolizumab (50), atezolizumab (38), nivolumab (3), durvalumab (2)	1° (N = 54, 58.1%) 2° (N = 39, 41.9%)	24 (25.8)	73.0 (73.0–74.0)
Melanoma	31 (21.4)	Nivolumab (3), pembrolizumab (28)	1° (N = 24, 77.4%) 2° (N = 7, 22.6%)	10 (32.3)	77.0 (74.0–79.4)
Kidney	6 (4.1)	Nivolumab (4), nivolumab-ipilimumab (1), atezolizumab (1)	1° (N = 1, 16.7%) 2° (N = 5, 83.3%)	2 (33.3)	72.5 (70.2–75.4)
Bladder	6 (4.1)	Atezolizumab (3), nivolumab (2), avelumab (1)	1° (N = 2, 33.3%) 2° (N = 4, 66.7%)	4 (66.7)	72.5 (70.2–74.8)
Breast	4 (2.8)	Atezolizumab (4)	1° (N = 4, 100%)	3 (75.0)	73.5 (n/a)
Liver	3 (2.1)	Atezolizumab (3)	1° (N = 3, 100%)	0 (0)	76.0 (n/a)
Larynx	1 (0.7)	Nivolumab (1)	2° (N = 1, 100%)	0 (0)	70.0 (n/a)
Mesothelioma	1 (0.7)	Nivolumab-ipilimumab (1)	2° (N = 1, 100%)	0 (0)	75.0 (n/a)

**Table 2:** Patients with adverse effects leading to either permanent immunotherapy cessation or treatment interruption (*n* = 24).

Adverse Effect	Number of Patients (% of Whole Population)	Median Time to Side Effect (Months) (95% CI)	Grade ^1^ (% of the Affected Population)
Hepatitis	8 (5.5)	1 (1–5)	2 (37.5), 3 (62.5)
Pneumonitis/hemoptysis	7 (4.8)	5 (1–12)	2 (42.8), 3 (14.3), 4 (28.6), 5 (14.3)
Colitis	2 (1.4)	10 (n/a)	3 (100)
Hypophysitis	2 (1.4)	9.5 (n/a)	2 (50), 3 (50)
Nephritis	2 (1.4)	7.5 (n/a)	2 (50), 3 (50)
Dermatitis	1 (0.7)	1 (n/a)	4 (100)
Encephalitis	1 (0.7)	1 (n/a)	3 (100)
Vasculitis	1 (0.7)	8 (n/a)	3 (100)

^1^ As per Common Terminology Criteria for Adverse Events.

## Data Availability

The original contributions presented in the study are included in the article, further inquiries can be directed to the corresponding author.
